# Adolescent-parent interactions and communication preferences regarding body weight and weight management: a qualitative study

**DOI:** 10.1186/1479-5868-7-16

**Published:** 2010-02-16

**Authors:** Vanessa A Shrewsbury, Lesley A King, Libby A Hattersley, Sarah A Howlett, Louise L Hardy, Louise A Baur

**Affiliations:** 1 Physical Activity Nutrition and Obesity Research Group, Sydney University, Sydney, Australia; 2 Discipline of Paediatrics and Child Health, Sydney University, Sydney, Australia

## Abstract

**Background:**

This study aimed to canvass the nature of adolescent-parent interactions about weight, particularly overweight, and to explore ideas of how to foster supportive discussions regarding weight, both in the home and with family doctors.

**Methods:**

A market research company was contracted to recruit and conduct a series of separate focus groups with adolescents and unrelated parents of adolescents from low-middle socio-economic areas in Sydney and a regional centre, Australia. Group discussions were audio recorded, transcribed, and then a qualitative content analysis of the data was performed.

**Results:**

Nine focus groups were conducted; two were held with girls (n = 13), three with boys (n = 18), and four with parents (20 mothers, 12 fathers). Adolescent and parent descriptions of weight-related interactions could be classified into three distinct approaches: indirect/cautious (i.e. focus on eating or physical activity behaviors without discussing weight specifically); direct/open (i.e. body weight was discussed); and never/rarely discussing the subject. Indirect approaches were described most frequently by both adolescents and parents and were generally preferred over direct approaches. Parents and adolescents were circumspect but generally supportive of the potential role for family doctors to monitor and discuss adolescent weight status.

**Conclusions:**

These findings have implications for developing acceptable messages for adolescent and family overweight prevention and treatment interventions.

## Background

The nature and quality of adolescent-parent interactions has the capacity to positively or negatively influence adolescent psycho-social development [1], engagement in health risk behaviors [2], and disease management outcomes [3]. While weight management may not be the most pressing of issues that adolescents would like to discuss with their parents [4], overweight and obesity are highly prevalent and have serious health consequences in both adolescents [5,6] and adults [7]. Therefore, it is likely that discussions about weight management may arise in families.

Adolescence is a critical intervention point for the prevention and treatment of overweight [8]. Yet adolescent weight outcomes have been modest in lifestyle-based overweight prevention and treatment, which highlights the scope for improving interventions [9,10]. Although such interventions may be directed exclusively towards adolescents, many also involve parents who can be considered important players in supporting adolescents to adopt and maintain healthy lifestyle behaviors [11,12]. Optimizing adolescent-parent interactions around adolescent weight management and its related behaviors may be an important target in improving outcomes in future interventions. However, there are limited published data on the nature of adolescent-parent interactions and communication preferences around this subject.

In addition to their parents and family members, adolescents also consider health professionals as people they would turn to if they had concerns about their weight [13,14]. Yet studies of doctor-parent-child communication have typically focused on doctor-parent interactions [15], thus consideration of adolescent preferences for doctor-parent-adolescent interactions in the context of weight management is warranted.

This study aimed to canvass both adolescent and parent views on adolescent-parent interactions around body weight, particularly overweight, and their opinions as to how body weight can be discussed in a positive and supportive way at home, and with the family doctor. This study focused on adolescents and parents from low to middle socioeconomic groups as they tend to be at higher risk of overweight [16].

## Methods

### Study design

A qualitative study design involving focus groups was selected as an appropriate method for an initial exploration of both adolescent and parent views on weight-related interactions and the breadth and strength of their publicly expressed attitudes [17]. The present study was the third and final phase in a larger focus group study, involving the same participants, that also sought to examine adolescent-parent interactions around common behaviors among adolescents that are associated with overweight development i.e. screen time and sugary drink intake [18]. An accredited market research company (MRC) was contracted by the research team to: i) recruit participants from their database; ii) facilitate focus groups; and iii) transcribe audio recordings. In all phases the research team maintained close relations with the MRC. The study was approved by the Ethics Committees of The Children's Hospital at Westmead and The University of Sydney.

### Participants and recruitment

Detailed information about participant eligibility criteria and the recruitment process is reported elsewhere [18]. Briefly, adolescents in grades 8-10 and unrelated parents or guardians of that age group were recruited from areas classified as low to middle socio-economic status (SES) in the Sydney metropolitan area and a regional centre, New South Wales, Australia. Practical considerations meant that the researchers could not guarantee that the separate adolescent and parent focus groups would be held simultaneously. Therefore only one family member was eligible to participate in order to avoid response contamination. Participants were required to speak fluent English in order to be able to participate fully in the discussions. Weight status was not specified as an inclusion criterion to permit a broader scope of opinions to be heard on the weight topic (phase 3), as well as the two other study topics (prior study phases) which are of relevance to overweight prevention. Written informed consent to participate in the study was given by adolescents (and their parents) and unrelated parents.

### Focus group procedure

Nine focus groups were scheduled in community venues during March 2008 including: two female and three male adolescent groups; and four parent groups (i.e. mothers only, fathers only, and two combined mother/father groups). A third male adolescent focus group was scheduled because it was anticipated that male groups may yield less discussion than the female groups. Three focus groups were held in the regional centre and six groups were held in the metropolitan area. Focus groups involved between five and eight participants, were 90-120 minutes in duration, and were audio-recorded. A focus group discussion guide was developed to address the study aims in a way that would promote interest and open discussion (Appendix 1). The primary facilitator was from the MRC and a researcher from the study team (VAS) observed each group; following completion of nine focus groups both agreed that response saturation had been attained.

### Data analysis

Anonymized transcripts of the audio-recordings, typed verbatim, were checked for quality by a member of the research team. A qualitative content analysis of the transcripts was conducted. Three members of the research team independently read the transcripts and discussed the key ideas and common themes arising in the adolescent and parent focus groups. Following agreement on a draft coding structure, two members of the research team independently coded the data separately for the adolescent and parent focus groups. A third member of the research team checked the results for consistency and minor revisions were made to the coding structure where appropriate. Consensus on the final coded data was reached in a straightforward manner and summaries of the findings were checked by members of the research team for accuracy.

## Results

### Participant characteristics

The study eligibility criteria were met by 103 of the 402 people who responded to the initial study invitation with 63 people eventually participating in a focus group. Adolescents (n = 31; 42% female) from each school grade were equally represented. The unrelated parent participants (n = 32; 63% mothers) had a mean age of 44.9 years (SD: 5.8). Almost 20% of parent and 13% of adolescent participants were born outside Australia. Almost one-third of all participants lived in areas classified as low or low-to-middle SES, with the remainder of participants residing in areas ranked as middle SES. Further information on participant characteristics are published elsewhere [18]. Several participants in the adolescent and parent focus groups voluntarily spoke about their body size, and some parents described their adolescent's body size. The study team member who attended each group confirmed that participants had a range of body sizes.

### Summary of focus group discussions

#### Adolescents' and parents' perceptions of weight management attempts in their household

Adolescents described weight management practices used by parents, or parents' actions that influenced other family members, and to a lesser extent their own or siblings' practices. Adolescents typically described examples of self management strategies that involved making healthy lifestyle changes such as increasing physical activity and modifying food intake. Despite earlier discussions about screen time and sugary drinks [18] modification of these behaviors was not specifically raised by participants. Some adolescents mentioned that their mothers had sought assistance outside the home for weight loss through commercial weight loss programs or by talking with a doctor.

Parents typically gave examples of their own experiences in trying to lose weight, as well as those of their spouse or partner, and to a lesser extent those of their children. Self management for parents generally involved attempts to exercise more or to adopt weight loss diets. Other parents had consulted a doctor, a dietitian, or a commercial weight loss program. Some parents mentioned that they had taken their adolescent to a dietitian or a specialized weight management clinic. Unsafe or concerning weight loss practices (e.g., use of diet pills, skipping meals) were not raised by parents or adolescents.

#### Adolescent-parent interactions regarding adolescent weight status

Adolescent-parent interactions described by both adolescents and parents with regards to adolescent weight could be broadly classified into three approaches. Each approach is discussed in turn with illustrative quotes from the focus groups:

##### i) Indirect or cautious

Interactions of this type were characterized by a focus on eating and physical activity behaviors without specifically mentioning adolescent body weight or size. This type of interaction was most frequently described by adolescents and parents. According to some adolescents, interactions with their parents were a one-way process, with parents telling them that they 'should' be doing something such as cutting down on junk food. A female adolescent, who had consulted a health professional regarding weight management, offered the following deeper insight (but this was an exception): *"I think parents have those ways of softening what they really want to tell you, like by saying 'there's a new basketball team starting up, do you want to do that? I think you should do that, it would be good for you.' It's kind of their way of saying you're getting pudgy and you need to lose some weight. Often you know that but they'll try and sugar-coat it"*.

Generally, parents said that they gave suggestions to steer their adolescent into making healthy food choices or being more active. Sometimes this type of interaction was triggered when a perceived threshold for a particular behavior was breached. Some parents, who had concerns about their adolescent's weight, described a technique in which they initiated a discussion about weight with their adolescent by initially talking about someone else with a weight problem. A cautious approach was also taken by some parents due to a concern that focusing on an adolescent's weight may initiate an eating disorder. For example one mother said: *"... you've got to be careful... you don't want to be blunt and say 'pull yourself together, you know you've got to do this, and you've got to do that', because you don't want them to go the other way...because anorexia is a terrible thing and bulimia is a terrible thing..."*.

##### ii) Direct or open

Interactions of this type involved specific references to adolescent body weight or size. Adolescents' descriptions of such interactions with their parents generally indicated sensitivity on the parent's behalf as shown in this female adolescent's comment *"But when she (my mother) mentions something about my weight or how I'm eating it'll be out of concern, not 'you're getting fat' you know"*. However, another female adolescent recalled *"Because I don't go to (sports) carnivals and everything they always get up me and reckon I've put on weight"*. A male adolescent described how his father recommended that he gain weight *"My Dad always says I'm like a stick, and he says 'try and slow down on the training and get a bit of weight on', he thinks I'm too small"*.

During direct interactions with their adolescent, some parents appeared to be conscious of preventing overweight or weight gain, such as this mother who said *"Yes, see when it comes to recess - I do their lunch and fruit... I explain to her, just choose a treat, and she says why, and I say because if you have too many treats, like if you have a chocolate and this and that, you're only going to get fat, like where is it going to go?"*. However, mostly a direct and open approach was described by parents who perceived that their adolescent was overweight. Interactions described by parents were generally encouraging but some appeared unpleasant. In several groups, parents recalled that their adolescent's body weight was discussed because their child initiated the discussion, as this father recalled *"In my case my daughter brings it up. I don't bring it up because I don't think that she's got a weight issue...often actually it's her older sister that brings it up because her older sister is a bit thinner than she is so she calls her fatty... quite often it will come up through teasing or something"*.

##### iii) Never or rarely discuss weight as a topic

Some parents said that weight was never discussed because their adolescent was a normal weight or did not like talking about the subject. Similarly, some adolescents said that weight was never discussed.

#### Other types of verbal interactions about weight

Some parents were aware that their adolescent was teased about their weight, particularly by siblings. Two of the male adolescent groups, but neither female adolescent group, shared anecdotes about interactions with siblings or peers around the subject of body weight. The nature of these interactions appeared to be mostly playful teasing; for example a male adolescent said *"...my older sister said I look heaps better now I'm not fat. Then she says I'm still fat, just mucking around, and I call her fat too"*. Hurtful teasing was never acknowledged in the focus groups. In both female adolescent groups, but none of the male groups, negative interactions with grandparents around body weight were recalled. The style of conversation was generally consistent with the direct approach, as shown in this female adolescent's comment: *"My grandpa... he's like if I'm eating a chocolate bar, he'll be like don't eat that all at once, save some for later, you'll gain weight or whatever, and he'll be like you have to stay healthy and he'll remind my mum..."*.

#### Adolescents' opinions on supportive and helpful ways to talk about body size, particularly overweight, at home

Overall, adolescents had little to say on this subject. Adolescents thought it was important for parents to show sensitivity; for example a male adolescent said *".... it's the way you say it. If you're like, 'You're hell fat' or if you say, 'You're getting a bit fat' and be nice about it. It depends on how sensitive a person is, some people might say it hard, it depends on the person"*. Encouragement was also considered important as shown in this comment made by a male adolescent: *"Normally they (the adolescent) should know they need to do something about it (overweight) and they just need a push"*. Although adolescents were specifically asked about supportive and helpful ways to *'talk' *about body size, non-verbal strategies that parents could take were commonly suggested, such as the example given by this male adolescent *"Maybe actions might help, like putting more veggies in the dinner, you know, cooking better meals...or just say go on family walks or something, and then the message might sink in..."*.

#### What would parents do if their adolescent was overweight or struggling with their weight?

Parents responded to this discussion topic hypothetically, or by talking about their real life experiences. The vast majority of parents recommended self management strategies within the household as the initial approach to assist their adolescent with weight management. Typically these strategies encompassed one or more of the following dimensions:

##### i) Discussion with adolescent

Parents said they would have discussions about nutrition and/or physical activity, by encouraging the desired behavior rather than simply telling their adolescent what to do. Some parents suggested taking a consultative approach to the discussion, in order to empower the teenager and involve them in the decision making process; for example one mother said *"I think it's a matter of talking about those things, and then making the decision together about it - so you are not saying you know 'you can't do this, can't do that' you've got to get them at this kind of age to make a choice themselves, because if they can start making that choice, then they are being responsible for themselves"*. In the fathers' group, it was the general consensus that they felt comfortable talking about weight issues with their sons but that it is preferable for mothers to have similar conversations with daughters.

##### ii) Home environment modification

Parents from all groups made suggestions for modifying their adolescent's food environment as a strategy to assist their adolescent with weight management. Some parents also said they would monitor and control their adolescent's food intake. Fewer parents suggested modifying the home physical activity environment.

##### iii) Participate in physical activity with their adolescent

Parents from all groups suggested that they would do more physical activities with their adolescent. Some parents specifically highlighted the importance of leading by example. However, in saying this, several parents acknowledged the practical difficulties in being able to be physically active with their adolescent.

##### iv) Other strategies

Some parents suggested building their adolescent's self-esteem as a strategy for assisting with weight management. As few parents across the groups mentioned that they would seek weight management assistance for their adolescent from outside the home, the facilitator prompted a discussion about the reasons for this. Seeking help was viewed as a last resort by parents, usually after one's own strategies had been exhausted for example one parent said *"If they (adolescents) were doing lots of exercise and they were eating well and it wasn't working then you'd be looking for another solution"*. Other parents thought consulting a health professional was only necessary if the adolescent's weight problem had become 'severe' and was affecting their child's physical or psychological health. Parental skills and confidence to assist adolescents with weight management were discussed in two groups. These parents reported that they had enough general knowledge to assist their adolescent with weight management, but that they might struggle with putting their knowledge into practice because of busy lifestyles.

#### Adolescents' and parents' opinions on family doctors routinely assessing an adolescent's weight status at check ups

The proposed idea for family doctors to routinely measure adolescents' height and weight and discuss adolescent weight status was largely supported by parents and adolescents, although this was highly conditional. Adolescents and parents highlighted the importance of doctors discussing adolescent body weight sensitively, particularly when adolescents are overweight. Some adolescents said this type of assessment may be embarrassing or make them feel bad and they speculated that overweight adolescents may be even more inclined to feel offended. For example one female adolescent said *"I don't think I'd mind very much but it's just embarrassing if you're overweight or not exercising enough. It's kind of embarrassing because you like don't even know this person very well and it's like they're pretty much telling you you're fat"*. Adolescents felt there were active steps that doctors could take to make young people feel more comfortable during this type of assessment, such as discussing the purpose of the measurements, mentioning that it was a routine procedure, and providing advice on nutrition and physical activity that meets the adolescent's individual needs.

When prompted, few parents could suggest how doctors might discuss overweight in a way that would be helpful and supportive to adolescents. However, concern over the potential to create weight concerns or initiate eating disorders in adolescents was mentioned in several parent groups. Some parents felt that weight should only be discussed if the young person was overweight, such as described by this mother *"I'm not saying that you ignore it if it is a problem but maybe if there's a borderline and we're going to put them on a scale and say, 'You look fine but actually on paper you're not' and suddenly they're 'Oh, I've got a weight issue and I didn't know I did'*. Other parents opposed the idea of routine weight assessments on the grounds that parents already know if their adolescent is overweight. Parents felt that it was important to consider the adolescent's age as to whether the adolescent and/or parent are informed about the doctor's assessment, although generally parents wanted to be involved. In the adolescent groups, opinions on parents being informed of adolescents' measurements also varied, with many adolescents wanting control over who was informed about the outcome of assessments.

## Discussion

In this study, weight related discussions between adolescents and their parents had taken place in many of the participants' households. Overall there was remarkable concordance between the views expressed by adolescents and parents. One of the main findings was that indirect communications between adolescents and parents around adolescent body weight were commonly used by parents and preferred by adolescents. This involved a focus on behaviors or actions associated with weight but not weight itself. While some participants did not recall weight discussions taking place at all, others described direct or open discussions, where body weight or size was specifically talked about.

There was an overarching sense that parents and adolescents in these focus groups were interacting in a mutually acceptable way around the subject of adolescent body weight. While some parents specifically said that they would endeavor to 'encourage' their adolescent in weight-related behaviors, adolescents tended to perceive parents as directive. This discrepancy may reflect typical differences between adolescent and parent perspectives. The fine tuning of adolescent-parent interactions (discussing versus telling) is an important parenting skill that could be further promoted in interventions targeting healthy weight management in adolescents.

Although the focus group discussion guide for the adolescent group specifically probed about their ideas on helpful and supportive ways for parents to 'talk about' adolescent overweight, adolescents commonly gave examples of non-verbal actions that their parents could take. Again, this is consistent with the idea that indirect approaches to discussing body weight are preferred by adolescents. Findings from Project EAT showed that parents who recognized their adolescent as overweight, compared with those who did not, were more likely to encourage their child to diet; in fact this was associated with increased risk of sustained overweight five years later [19]. The study authors concluded that "instead of focusing on weight *per se*, it may be more helpful to direct efforts towards helping parents provide a home environment that supports healthful eating, physical activity, and well-being". The present study lends support to these recommendations. However, given that parents frequently do not recognize overweight in their children [20], the avoidance of direct discussions may sometimes reflect defensiveness on the part of family members, and in some cases be counterproductive.

While parents had a good knowledge of general self-management strategies for supporting adolescent weight management, they may need help with putting knowledge into practice. Parents in this study were reluctant to involve healthcare professionals unless an adolescent's weight problem became severe and their physical or psychological health noticeably deteriorated. Similarly, in a qualitative study involving parents who had concerns about their child or adolescent's weight, all parents had tried to manage their child's persistent weight gain before seeking professional help [21]. Therefore, raising parents' awareness about the benefits of early intervention is important and further research is needed around the strengths and weaknesses of self versus professional management of child and adolescent overweight.

Although family doctors have limited time to discuss weight management with individual patients [22] they may be the best placed professionals to assess and monitor weight. In this study, adolescents and parents largely supported the proposal for family doctors to routinely measure adolescent height and weight and discuss weight status, but the need for doctors to be sensitive in doing this was emphasized. Our findings are consistent with those of a quantitative study in which approximately two-thirds of caregivers responded that a physician's concern about a child's weight should be discussed with the child present, as long as it was talked about sensitively [23]. In that study, one-sixth of parents were concerned that discussing weight with a child may increase the risk of eating disorders or lowering a child's self-esteem. Initiating an eating disorder or weight issues in adolescents was also a concern for some parents in the present study. These parental concerns should be considered with other factors [24] when clinicians discuss weight status with adolescents.

The present study successfully involved a market research company to recruit participants from low to middle SES neighborhoods in both a metropolitan area and a regional centre. A strength of this study is that we examined perceptions of adolescents, mothers and fathers around adolescent-parent interactions on the subject of body weight. To explore more detailed aspects of adolescent-parent interactions, we would recommend that this topic is further explored using other study designs such as adolescent-parent dyad interviews. As the participants were predominately Australian born and their weight status was heterogeneous, the findings may not be generalizable to other ethnic or specific weight status groups. Future research on this topic is recommended in those sub-groups.

## Conclusions

The implications of this research for clinical practice are that if an indirect approach to discussing weight management is preferred by adolescents and parents, then health professionals and researchers should consider emphasizing behavioral rather than weight outcomes in the recruitment, intervention, and assessment phases of adolescent weight management programs. Parents may also benefit from professional assistance to develop supportive indirect communications with their adolescent children.

## Competing interests

The authors declare that they have no competing interests.

## Authors' contributions

VS, LK, LHatt, LH and LB all contributed to the development of research questions and the design of the study, and the Ethics Committee submission. VS, LK and LHatt were involved in contracting the market research company and providing specifications and protocols for recruitment and conduct of groups. VS attended all focus groups and checked all transcripts. VS, LHatt, LK and SH undertook data coding and initial analyses; LH, LB, LK and SH were involved in checking the coding and analysis. All authors contributed to the interpretation of data and the writing of the manuscript. All authors read and approved the final manuscript.

## Appendix 1. Focus group discussion guide

*Introduction: Both underweight and overweight can be a health concern, however today's discussion will focus on overweight and weight management*.

1. What kinds of things have people in your household done to manage their body weight in the past year?

2. How openly is weight and weight management discussed in your household?

3. (*Adolescents only*) What kinds of things to do with body size have you talked about with your mum, dad, brothers or sisters?

4. (*Parents only*) What do you think your teenager thinks about their body size and why do you think this? What kinds of things might be discussed about your teenager's body size between you and your teenager, or your partner and your teenager? If your teenager was struggling with their weight what would you do?

5. What are your thoughts about family doctors measuring the height and weight of teenagers routinely during a visit so that doctors could let teenagers know whether their weight was in the healthy, overweight or underweight range for their age?

6. How can body size, especially overweight, be talked about in a supportive and helpful way at home and with health professionals?
